# Textural heterogeneity of liver lesions in CT imaging - comparison of colorectal and pancreatic metastases

**DOI:** 10.1007/s00261-024-04511-5

**Published:** 2024-08-08

**Authors:** Friedrich L. Pietsch, Florian Haag, Isabelle Ayx, Freba Grawe, Abhinay K. Vellala, Stefan O. Schoenberg, Matthias F. Froelich, Hishan Tharmaseelan

**Affiliations:** 1 Department of Radiology and Nuclear Medicine, University Medical Center Mannheim, Heidelberg University, Theodor-Kutzer-Ufer 1-3, 68167 Mannheim, Germany; 2 DKFZ Hector Cancer Institute at the University Medical Center Mannheim, Im Neuenheimer Feld 280, 69120 Heidelberg, Germany

**Keywords:** Tumoral Heterogeneity, Radiomics, Colorectum, Pancreas, Tumor/Oncology, Computed tomography

## Abstract

**Purpose:**

Tumoral heterogeneity poses a challenge for personalized cancer treatments. Especially in metastasized cancer, it remains a major limitation for successful targeted therapy, often leading to drug resistance due to tumoral escape mechanisms. This work explores a non-invasive radiomics-based approach to capture textural heterogeneity in liver lesions and compare it between colorectal cancer (CRC) and pancreatic cancer (PDAC).

**Materials and methods:**

In this retrospective single-center study 73 subjects (42 CRC, 31 PDAC) with 1291 liver metastases (430 CRC, 861 PDAC) were segmented fully automated on contrast-enhanced CT images by a UNet for medical images. Radiomics features were extracted using the Python package Pyradiomics. The mean coefficient of variation (CV) was calculated patient-wise for each feature to quantify the heterogeneity. An unpaired t-test identified features with significant differences in feature variability between CRC and PDAC metastases.

**Results:**

In both colorectal and pancreatic liver metastases, interlesional heterogeneity in imaging can be observed using quantitative imaging features. 75 second-order features were extracted to compare the varying textural characteristics. In total, 18 radiomics features showed a significant difference (*p* < 0.05) in their expression between the two malignancies. Out of these, 16 features showed higher levels of variability within the cohort of pancreatic metastases, which, as illustrated in a radar plot, suggests greater textural heterogeneity for this entity.

**Conclusions:**

Radiomics has the potential to identify the interlesional heterogeneity of CT texture among individual liver metastases. In this proof-of-concept study for the quantification and comparison of imaging-related heterogeneity in liver metastases a variation in the extent of heterogeneity levels in CRC and PDAC liver metastases was shown.

**Supplementary Information:**

The online version contains supplementary material available at 10.1007/s00261-024-04511-5.

## Introduction

Despite modern and personalized therapies, gastrointestinal cancers remain highly prevalent worldwide [1]. Colorectal cancer (CRC) is the third most diagnosed cancer, with over 1.9 million new cases and 935,000 deaths worldwide in 2020 [2, 3]. Pancreatic cancer (PDAC), while not as prevalent, remains one of the most lethal malignancies, characterized by subclinical onset, aggressive progression, and poor prognosis. In 2020, it accounted for 496,000 new cases and 466,000 deaths worldwide, ranking as the 12th most common malignancy [4]. In recent years, survival rates have been improved for CRC patients due to increased prevention measures (e.g. colonoscopy, endoscopic ultrasound, or MRI) and improved therapies for both colorectal and pancreatic cancer [5, 6].

Nevertheless, patients frequently present in the advanced stage of disease. Approximately 15-30% of CRC patients present with metastases at initial diagnosis. The five-year survival rate drops from 90.9% at the localized stage to 15.6% in the metastatic stage [5]. In comparison, 51% of PDAC patients present with metastases at the initial diagnosis [6]. This contributes to low overall survival rates of 12.5% [6], 42.2% for localized, and 5.9% for distant stages [7].

Both gastrointestinal tumors predominantly metastasize to the liver, with approximately 30–50% of metastases occurring in this organ. [8] They present as hypodense liver metastases with similar features in radiological imaging. However, CRC and PDAC differ significantly in their aggressiveness, making a comparison of their characteristics on imaging essential for understanding their shared and unique characteristics.

The management of colorectal and pancreatic cancers is subject to many challenges. For example, the long time to detection and the lack of adequate treatment options in advanced stages are major hurdles. In terms of the personalization of therapy to improve outcomes, the heterogeneous nature of CRC and PDAC is a highly relevant factor [9–12]. The individual expression of proliferation rates, the tumoral microenvironment, metabolic activity, vascularity, cell death, and other characteristics necessitate targeted adaptation. Colorectal and pancreatic tumors exhibit histological and genetic variability not only among multiple lesions in a single patient (interlesional heterogeneity) but also within a single lesion (intralesional heterogeneity) [13, 14]. Spatial heterogeneity manifests as regional differences within the tumor at one point in time. In contrast, temporal heterogeneity describes fluctuations and alterations in these features throughout disease progression or in response to treatments, reflecting dynamic changes [15, 16].

The presence of tumoral heterogeneity is linked to unfavorable tumor biology [12, 15, 17]. Determination of heterogeneity by individual biopsies can be challenging, as these often cover a small part of the present variability within single and multiple lesions at the time point of resection. Therefore, focusing on heterogeneity as a tumoral escape mechanism impeding the targetability of lesions may improve personalized cancer treatment. The characterization of tumoral heterogeneity has primarily focused on molecular and cellular biology, with comparatively less emphasis on its description through imaging modalities.

In clinical oncology, imaging plays a fundamental role in lesion characterization, staging, treatment planning, and therapy monitoring [18]. While for many entities Positron emission tomography (PET) or Magnetic resonance imaging (MRI) are used more often [19], Computed tomography (CT) remains essential for primary staging [20, 21]. One drawback common to all imaging modalities is that interpretation is limited by the visual capabilities of the reading radiologist. Subtle nuances not perceivable by the human eye, that may be frequently missed can be therapy-relevant and important prognostic factors. While traditional image-based analysis can capture characteristics such as signal intensity or Hounsfield Units (HU), it does not account for spatial distribution, making it impossible to comprehend the complexity of a heterogeneous pattern.

The satisfactory detection of tumoral heterogeneity through conventional imaging and assessment by a radiologist remains challenging. However recent years have seen an increase in quantitative image analysis, particularly in the form of radiomics, which attempts to address this challenge [22]. Radiomics serves as a promising methodology for the extraction of comprehensive quantitative data, encompassing non-interpretable image and texture features obtained from radiological imaging [23]. The technique explores the structure of tumors, offering a quantification that captures the extensive heterogeneity within a tumor, potentially revealing details that are not detectable by visual analysis [24].

Radiomics has already been utilized to describe intralesional heterogeneity in single lesions of colorectal cancer [24, 25]. Henry et al. characterized intertumoral intrapatient heterogeneity in lung cancer metastases and melanoma metastases through the extraction of radiomics features, using the coefficient of variation (CV) as a quantitative parameter [26]. Radiomics has also been evaluated in its detection of heterogeneity in pancreatic ductal adenocarcinoma (PDAC), with studies demonstrating its potential in predicting overall survival and improving prognostic accuracy compared to traditional staging methods. [27–29] However, to date, there has been no comparison between heterogeneity in liver metastases, originating from two different gastrointestinal cancer diseases. This study aims to evaluate interlesional intrapatient heterogeneity by quantifying imaging characteristics in colorectal and pancreatic liver metastases, utilizing radiomics analysis of computed tomography scans.

## Methods

### Patient collective

**Fig. 1:**
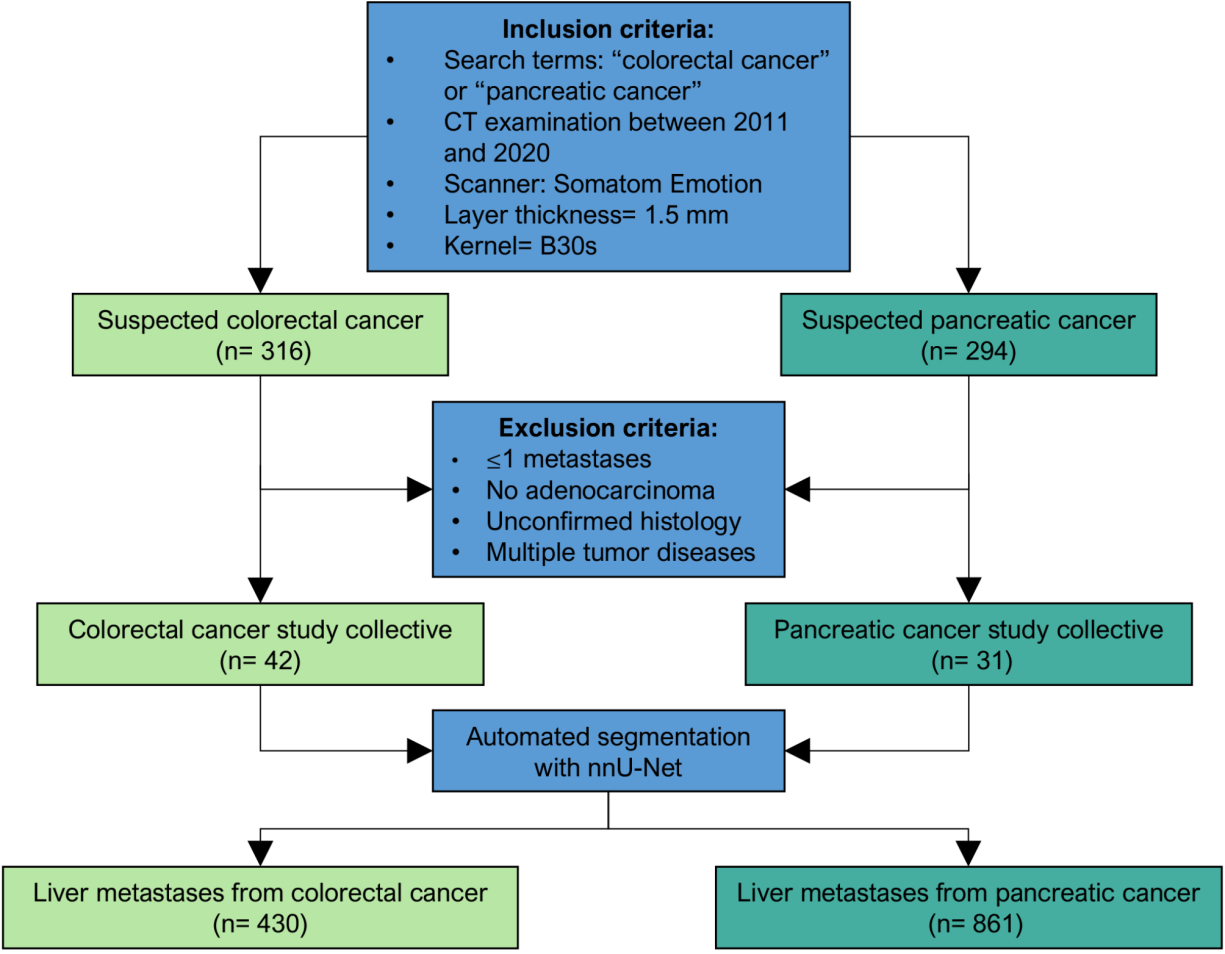
Flow diagram summarizing patient collective and selection criteria

A retrospective search query from an institutional database, specified by the terms “colorectal cancer” and “pancreatic cancer”, identified CT examinations conducted between 2011 and 2020. Patients with solitary liver metastases, other histology than colorectal / pancreatic ductal adenocarcinoma, or multiple tumor diseases and unconfirmed histology were excluded. The selection criteria are summarized in Fig. 1.

### Imaging protocols

All patients diagnosed with CRC and PDAC received staging in a 16-slice abdominal contrast-enhanced CT (Siemens Somatom Emotion, Siemens Healthineers, Erlangen, Germany). All scans were performed using B30s kernel reconstruction with 1.5 mm slice thickness in axial orientation and portal venous contrast enhancement phase (60 s delay). The applied contrast agent was 90 ml of a non-ionic, monomeric X-ray intravenous contrast agent Imeron^®^ (Bracco Imaging, Milan, Italy) with a 2.5 ml/s flow.

### Liver and lesion segmentation

The liver and existing lesions underwent fully automated segmentation through the nnUNet using the non-customized and pre-trained model specific for liver and liver tumor segmentation [30]. Subsequently, the metastases segmentation mask was split into individual lesion masks in 3DSlicer (version 4.11.20210226). If the region of interest (ROI) could not be detected satisfactorily in the first step, a correction of the identified areas was then carried out manually. A clinical radiologist (M.F.F.) reviewed the resulting segmentations. Lesions smaller than 0.5 cm were excluded from further analysis.

### Radiomics feature extraction and selection

75 Radiomics features were extracted within each segmented liver lesion by employing Pyradiomics (version 3.0.1) in Python [31]. To provide insights into the heterogeneity of lesions, we focused on the texture and only extracted second-order features such as gray level co-occurrence matrix (GLCM), gray level run length matrix (GLRLM), gray level size zone matrix (GLSZM), gray level dependence matrix (GLDM), and neighboring gray tone difference matrix (NGTDM), which describe inter-relationships between neighboring voxels and measurements of the spatial arrangement of voxels [32].

### Quantification of heterogeneity

The following statistical analysis was performed using R in RStudio (version 1.3.1093, Boston, MA, USA). To measure the heterogeneity between different lesions, the coefficient of variation (CV) was calculated for each radiomics feature. The CV, representing the ratio of standard deviation (σ) to mean (µ), functioned as a precise measure of the variation in particular radiomics features.

$$\:\text{C}\text{V}=\frac{{\sigma\:}}{{\mu\:}}\:\cdot\:100\text{\%}\:$$

Higher CV values indicate increased variability, signifying heterogeneity between the lesions within a patient (interlesional heterogeneity). The utilization of CV as a quantitative parameter enabled a more precise understanding and assessment of the varying levels of heterogeneity present in the texture of both malignancies. Heatmaps derived from the CV of each feature value were generated patient-wise to represent the deviating heterogeneity patterns visually.

### Comparison of heterogeneity between colorectal and pancreatic liver metastases

**Fig. 2:**
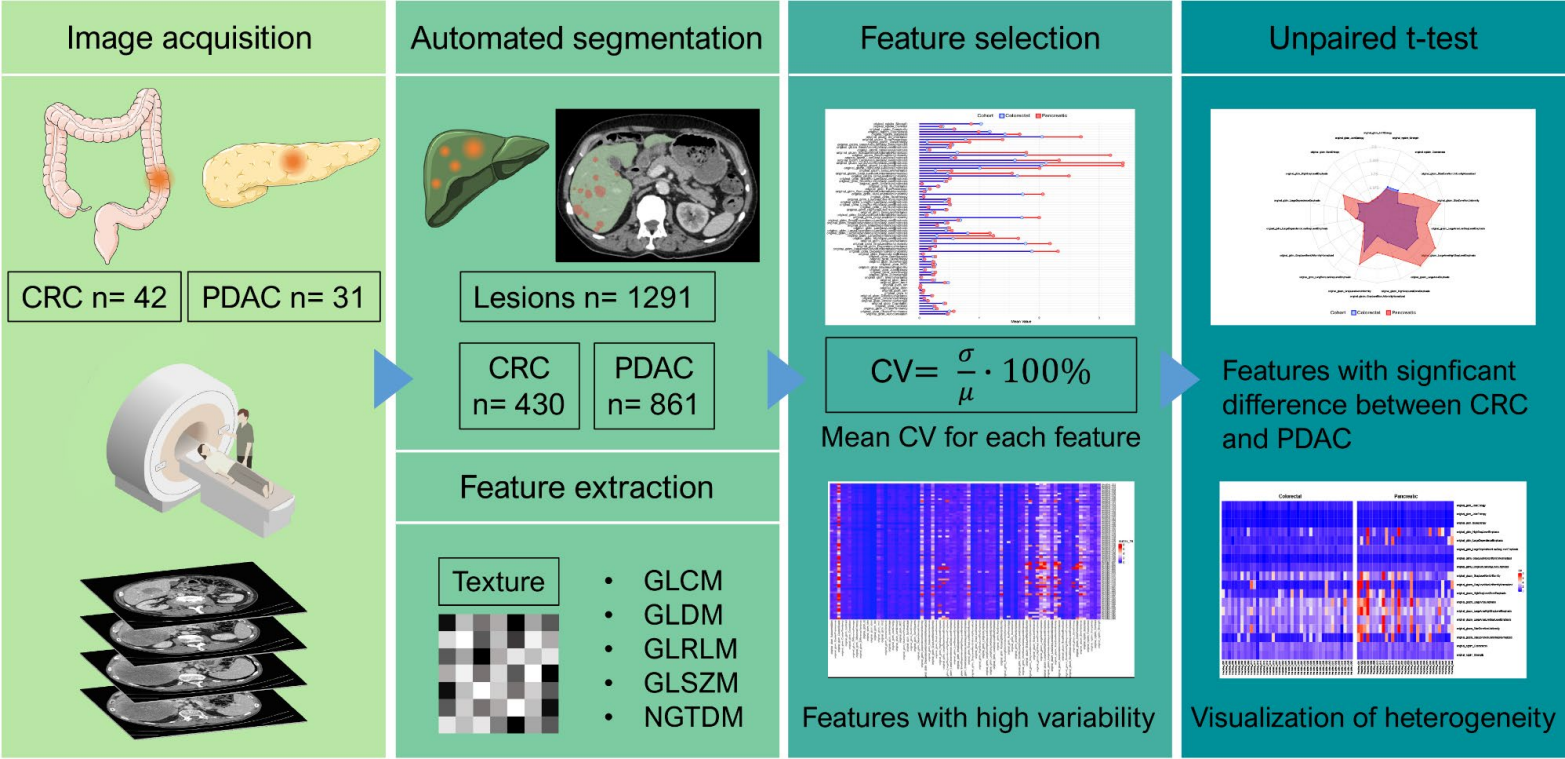
All 42 colorectal cancer (CRC) and 31 pancreatic cancer (PDAC) patients were examined in Siemens Somatom Emotion CT. Lesions were segmented fully automated using the nnUNet, and the regions of interest (ROI) were manually corrected in 3D Slicer. Radiomics features were extracted in Python through the application of the Pyradiomics package. The features demonstrating a statistically significant difference in coefficient of variation (CV) and thus high variability between both groups were identified by applying an unpaired t-test

To compare the interlesional radiomic heterogeneity of the liver metastases between patients with CRC and PDAC, an unpaired t-test was applied to identify significant differences in feature variability, utilizing the CV as the comparative metric. The CV was calculated based on the heterogeneity of lesions within a patient for each radiomics feature. The following application of the unpaired t-test on the resulting CV values then allowed the comparison between both cancer groups. Radiomics features, demonstrating significant disparities through the t-test, highlighted distinct variations between liver lesions of metastatic CRC and PDAC, indicating differing radiomics profiles among the studied tumor types. An alpha level of 0.05 determined statistical significance. A radar plot was created to visualize the significant features, providing an illustration that compares the extent of heterogeneity in the CRC and PDAC metastases. The analysis workflow is summarized in Fig. 2.

## Results

### Patient collective and lesion segmentation

Based on the criteria, 42 CRC patients and 31 PDAC patients were included. In the CRC collective, 66.7% of the patients were male and had a median age of 65.5. In comparison, in the PDAC collective 48.4% of the patients were male and had a median age of 65. Overall, 430 liver metastases from colorectal carcinoma and 861 metastases from pancreatic cancer could be identified. On average CRC patients had 10.24 liver lesions and PDAC patients showed 27.77 liver lesions. The patient characteristics are summarized in Table 1.

**Table 1:** Patient characteristics. To specify values for the size range, the feature “original_shape_VoxelVolume” was used for approximation. From the data for each lesion of each patient, the average lesion size for the cohorts and the *p*-value were calculated using a Wilcoxon-Mann-Whitney test

		Colorectal Cohort	Pancreatic Cohort	*P*-Value
**n**		42	31	
**Age (median [IQR])**		65.5[54.5, 70]	65 [56, 74.5]	0.4617
**Sex (%)**	*F*	14 (33.3%)	16 (51.6%)	0.1508
	*M*	28 (66.7%)	15 (48.4%)	
**T-Stage (%)**	*T1*	2 (4.8%)	1 (3.2%)	0.07582
	*T2*	4 (9.5%)	3 (9.7%)	
	*T3*	21 (50.0%)	6 (19.4%)	
	*T4*	13 (31.0%)	19 (61.3%)	
	*Tx*	2 (4.8%)	2 (6.5%)	
**N-Stage (%)**	*N0*	7 (16.7%)	6 (19.4%)	0.8339
	*N1*	16 (38.1%)	11 (35.5%)	
	*N2*	18 (42.9%)	14 (45.2%)	
	*Nx*	1 (2.4%)		
**M-Stage (%)**	*M1*	42 (100.0%)	31 (100%)	1
**Liver lesions**	*Number*	430	861	0.0004878
	*Mean*	10.24	27.77	
	*Min.*	2	2	
	*Max.*	42	85	
	*Median*	6,5	15	
	*Size Range (mean)*	30311.06	5703.75	0.03227

### Coefficient of variation analysis

**Fig. 3:**
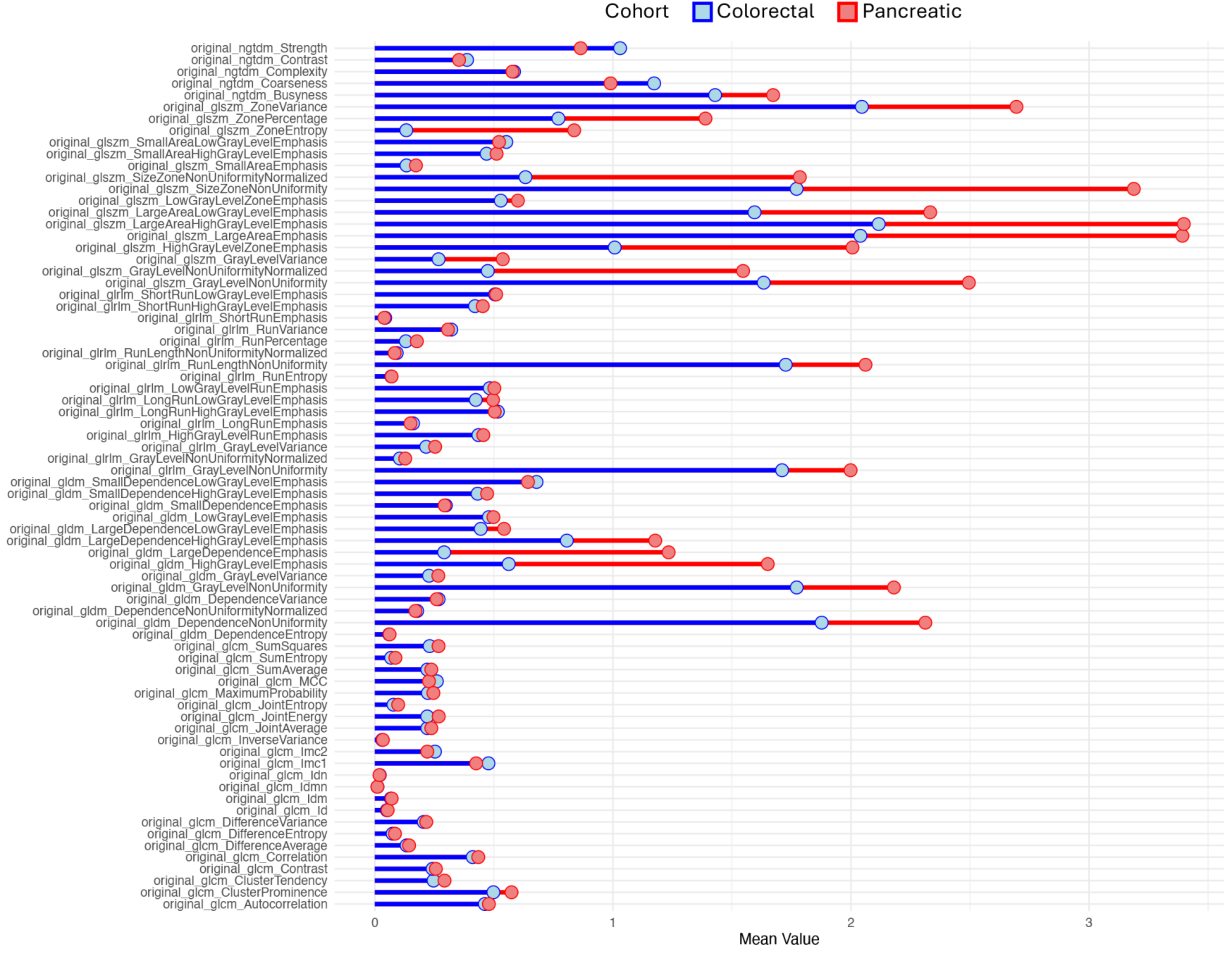
Lollipop diagram showing the mean CV for colorectal and pancreatic cases, with all features except “original_glcm_ClusterShade”

The mean CV for each radiomics feature was computed and displayed in a lollipop plot (Supplemental Fig. S1) for each cohort, with the calculation incorporating the values from all metastases (CRC vs. PDAC). The feature “original_glcm_ClusterShade” was showing an extreme level of variance compared to other features (mean CV of 25–40 vs. other features ranging from 0 to 3.5) in both cohorts. To improve the comparability of all other features, a second plot without the Feature “original_glcm_ClusterShade” was created (Fig. 3).

### Feature selection by CV

**Fig. 4:**
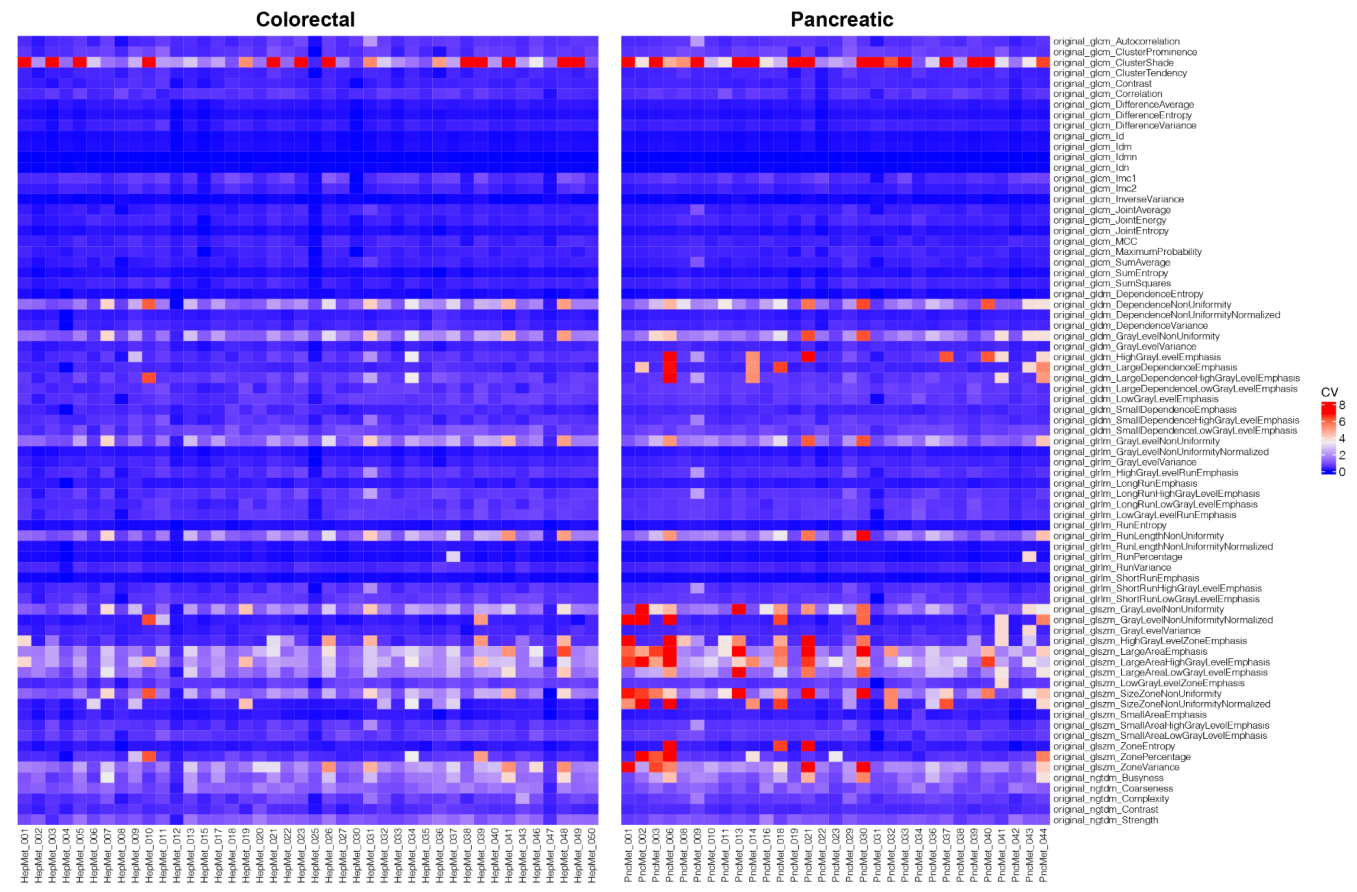
Unclustered heatmap visualizing the coefficient of variation (CV) value for each feature and patient

**Fig. 5:**
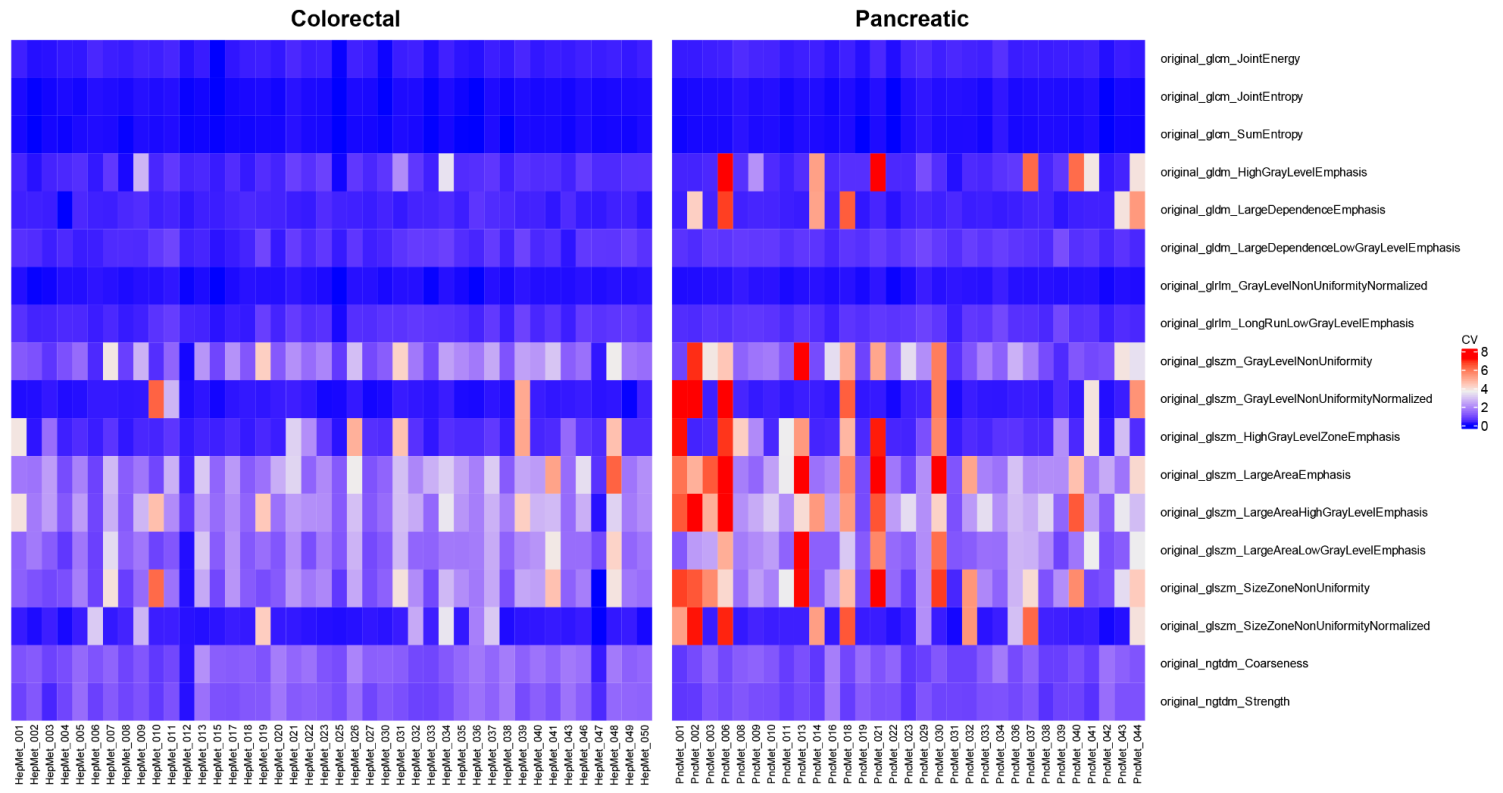
Unclustered heatmap with the final feature set of statistically significantly differing features (*p* < 0.05)

**Fig. 6:**
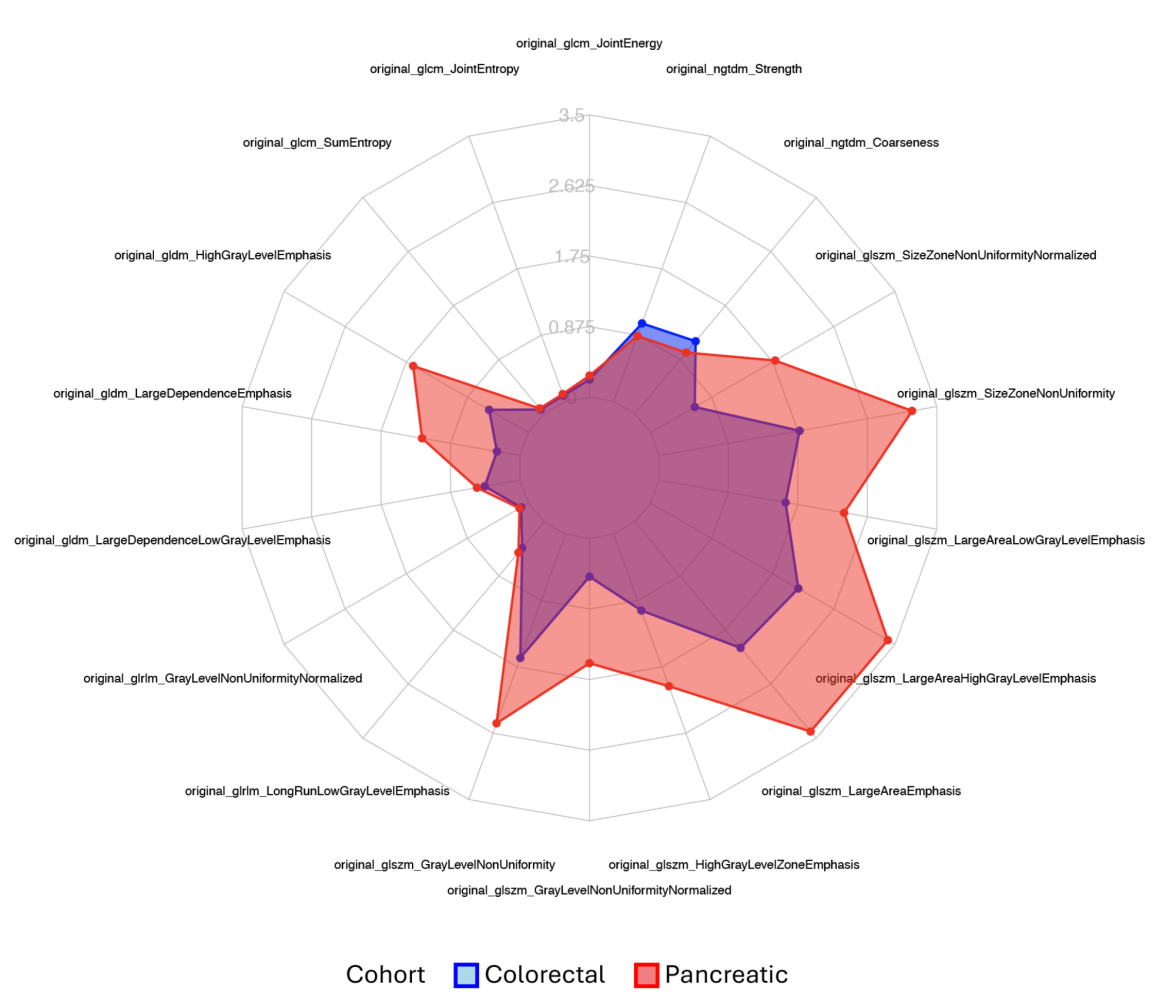
Radar plot for all significant features visualizing the higher level of heterogeneity in pancreatic metastases

The visual analysis of the created heatmap showed a differing level of variability for each feature with a high range of similar features (Fig. 4). The feature selection resulted in 18 features differing significantly (*p* < 0.05) between both groups. The features and respective boxplots are listed in supplemental materials (S2 and S3). The feature “original_glcm_ClusterShade”, which showed high variances of the means due to particularly high CV values, did not exhibit statistically significant differences. The final set of significantly differing features was displayed in a heatmap (Fig. 5) and a radar plot (Fig. 6). 16 radiomics features showed higher mean CV values in the PDAC subgroup compared to the colorectal cancer subgroup. The features “original_ngtdm_Coarseness” and “original_ngtdm_Strength” have a slightly higher CV value for the CRC subgroup. This indicates a higher variability of the tissue texture and thus a more pronounced interlesional radiomic heterogeneity in PDAC liver metastases compared to the liver metastases of CRC.

## Discussion

This study investigated the quantification and comparison of interlesional intrapatient heterogeneity in liver metastases using radiomics features. 75 second-order features in 1291 liver metastases were extracted and the heterogeneity was quantified by calculating their mean CV within each patient. 18 of the 75 intrapatient heterogeneity describing features showed significant variations in their expression levels when compared between patients with CRC or PDAC as their primary. Hence, using the 18 features, deviating textural patterns were identified, representing different levels of heterogeneity present in the metastases. Out of these, 16 features showed higher mean CV values in the metastases of the PDAC. At the level of CT imaging and evaluation through radiomics features, a higher degree of variability can be observed, which states a greater level of radiomic heterogeneity for this entity. However, two features exhibit greater heterogeneity in the CRC cohort. This also may suggest the presence of different dimensions of heterogeneity specific to the primary entity.

The higher degree of the textural radiomic heterogeneity in metastases of PDAC found in this work could be associated with the desmoplastic reaction of the tumor. Pancreatic adenocarcinoma generally exhibits a noticeable amount of fibrotic stroma. A large part of the tumor consists of heterogeneous cellular material, such as fibroblasts, immune cells, blood, and lymphatic vessels. The proportion of tumor cells can be less than 20% [33]. This is a characteristic of PDAC and not typical for CRC to this extent. The results of this work are in accordance with known patterns of metastases: The expression of heterogeneity is a possible representation of the specific nature of PDAC metastasis. The combination of different methods such as radiomics feature extraction and the parallel collection of biopsies and following genetic analysis of the material could provide more insights. A limitation of our study is the missing detailed histopathological or genetic data of the tumors, which is due to the retrospective study design. Previous studies, such as the work by Siravegna et al. have demonstrated significant underlying genetic heterogeneity in liver metastases through post-mortem biopsies. [34] The sensitivity of tumor clone growth and appearance to the local microenvironment, particularly in an organ like the liver, which depends on gradient structures such as blood supply, oxygen, and local signaling factors, could play a significant role. These factors might drive radiomic heterogeneity based on the tumor’s adaptability. Future studies should investigate the influence of the microenvironment on the radiomic characteristics of tumors in detail. Experimental approaches could help to better understand the relationship between histology, clonal growth, and radiomic heterogeneity. This inherent biological variability is an important factor that we cannot detect with our current dataset.

Schmelz et al. already demonstrated how the existence of spatial and temporal intratumoral heterogeneity can be evaluated through the genomic profiling of multiregional neuroblastoma biopsies [35]. Lohr et al. were able to emphasize the importance of heterogeneity analysis for treatment decisions by the sequencing of the genetic landscape in tumor samples of patients with multiple myeloma [36]. The integration of DNA profiling, serial tumor biopsies, and lesion-specific imaging can contribute to detecting mechanisms of drug resistance and guide therapeutic strategies [37].

However, for patients with an extensive metastatic spread, an accurate molecular analysis through single-lesion biopsies still can be challenging [38]. Although single sample collection is currently the standard for diagnosis, it frequently fails to identify molecular alterations that may be the cause of lesion-specific treatment failure [37]. A satisfactory comprehensive, especially non-invasive, approach is missing.

New imaging technologies, like photon-counting CT detectors, seem to offer promising opportunities for the application of radiomics feature analysis [39, 40]. Despite efforts to acquire the scans using one specific single-source CT device to ensure feature stability, a multicentric approach with more advanced detectors could provide interesting results. While aiming to quantify interlesional heterogeneity, we focused on the texture of metastases, extracting specific second-order features. However, the CT images, particularly from photon-counting detectors, offer the potential to derive data with higher resolution, hence providing more information for quantitative feature extraction. Additional subcategories, such as shape features that describe the geometric properties of the traced ROI, or first-order statistics features that depict the distribution of individual voxel values, could offer further insights [32]. Implementing advanced imaging technology could increase the accuracy and address issues related to radiomics feature stability [41]. The characterization of metastases by extracting these additional features could help to identify other pattern characteristics of the underlying primary source.

Liver metastases in general show high interlesional heterogeneity, especially compared to their primary [42]. Therefore, assessing genetic diversity and detecting acquired lesion-specific resistance mechanisms is a major objective of targeted drug therapy. Expanding the examination to include liquid biopsies or tumor samples can provide a more precise assessment [43]. Combining quantitative image data extraction and subsequent genetic analysis to validate the detected heterogeneity could help confirm the results. While the characterization of tumoral heterogeneity remains to rely on molecular and cellular biology, imaging modalities lately show great potential in this regard. The spotlight in personalized medicine has started shifting towards image-based analysis, aiming to offer non-invasive guidance to forecast treatment responses and survival durations [44]. Further research is necessary to detect different tissue textures reliably and establish a connection to the molecular causes.

Although the identification of interlesional intrapatient heterogeneity is a valuable insight, it is only a first step towards the identification of genetic alterations through imaging. Therefore, the results of this study contribute to a more precise assessment but must be considered in the context of its limitations.

The study is restricted to a small and reasonably homogeneous patient collective. In particular, the distribution of the analyzed lesions must be taken into account. Despite 42 patients being included in the CRC group and 31 in the PDAC group, the final number of liver metastases was higher in the pancreatic subgroup (430 CRC vs. 861 PDAC metastatic lesions). The distribution may reflect the specific metastatic patterns of the respective entity. While CRC patients presented an average of 10.24 liver metastases, PDAC patients exhibited 27.77 metastases. However, this results in an unequal distribution of extractable ground data. PDAC metastases showed a higher level of heterogeneity than CRC metastases. This may be the effect of the higher number of lesions per PDAC patient but could also be caused by an underlying presence of genetic interlesional variability and the desmoplastic reaction. Further analysis aimed at balancing the number of examined metastases could contribute to the validation of the observed differences in heterogeneity.

As this approach is based on the extraction of radiomics features, it is inherently sensitive to the influence of specific parameters relevant to this process. Fundamentally, the CT device and selection of imaging modalities influence the expression of the features [45]. Especially texture features are highly dependent on scanning parameters [46]. Thus, this study only comprised imaging data from a single CT scanner. To ensure the uniformity of data, only images with the same scanning parameters, a slice thickness of 1.5 mm, and B30s kernel reconstruction were included in the feature extraction and post-processing process [47, 48]. Feature stability can be influenced by various factors, including the region of interest, specifically its extent and size, as well as the following automated segmentation [49]. Subsequently, the segmentation results underwent correction and were reviewed by one clinical radiologist with experience in abdominal imaging, ensuring interrater reliability.

The CV was used as a statistical tool to quantify the heterogeneity. It should be emphasized that by applying CV a standardization was carried out, which made it possible to meaningfully compare the features with one another. Relative dispersion, commonly used to assess the extent of trait variation, offers the advantage of being dimensionless. When comparing variance among quantitative features by the mean CV, even basic statistics could lose relevance, if the context of the data is disregarded. This consideration is relevant because the analysis of radiomics features generally involves extensive data frames. Additionally, being dependent on the sample mean and standard deviation, outliers can have a negative impact on the calculation [50]. Moreover, the CV can only be used to compare data whose units are the same and whose domain does not contain negative data. Otherwise, correct calculation of the CV is sometimes not possible [51]. The basic requirements for the correct application have been taken into account.

While we acknowledge that the direct clinical relevance of radiomic feature variability between PDAC and CRC liver metastases may not be immediately apparent, our findings provide the groundwork for future studies. A following analysis including the treatment results and overall survival of the patients examined should be carried out. Previous research, such as the work by Sun et al. [52], has demonstrated that intralesional heterogeneity captured through radiomics can correlate with overall survival. This study showed that a radiomics signature of CD8 T-cells could predict lesion response and patient outcomes in melanoma patients treated with immunotherapy, highlighting the potential of radiomics to evaluate disease heterogeneity and patient prognosis. We believe that our study’s analysis of radiomics heterogeneity holds similar potential for developing prognostic markers in the future and expanding the included data for subsequent projects would be a valuable step forward.

In summary, this study serves as a proof of concept for comparing the heterogeneity between liver metastases in CT imaging across two gastrointestinal tumor entities, while also illustrating the interlesional radiomic heterogeneity among individual liver metastases originating from the same primary entity. Quantifying and improving the comparability of tumoral heterogeneity holds great potential to foster the tailoring of diagnostics and subsequent therapy.

## Electronic supplementary material

Below is the link to the electronic supplementary material.

Supplementary Material 1

## Abbreviations

CRC: Colorectal cancer
PDAC: Pancreatic ductal adenocarcinoma cancer
CV: Coefficient of variation
CT: Computed tomography
PET: Positron emission tomography
MRI: Magnetic resonance imaging
ROI: Region of interest
