# Supplementary material for: Textural heterogeneity of liver lesions in CT imaging - comparison of colorectal and pancreatic metastases
Source: Abdom Radiol (NY). 2024 Aug 8;49(12):4295–306. doi: 10.1007/s00261-024-04511-5 (PMC11522118; doi:10.1007/s00261-024-04511-5)
Supplement: Supplementary file 1 — Supplementary Material 1 [file 261_2024_4511_MOESM1_ESM.docx]

**Supplemental Material**

**Supplemental Material 1:** Lollipop diagram showing the mean coefficient of variation (CV) for all features (including “original_glcm_ClusterShade”) for both, colorectal and pancreatic cohort.

| Feature | p-value |
| --- | --- |
| original_glcm_JointEnergy | 0.0176 |
| original_glcm_JointEntropy | 0.0110 |
| original_glcm_SumEntropy | 0.0179 |
| original_gldm_HighGrayLevelEmphasis | 0.0166 |
| original_gldm_LargeDependenceEmphasis | 0.0158 |
| original_gldm_LargeDependenceLowGrayLevelEmphasis | 0.0073 |
| original_glrlm_GrayLevelNonUniformityNormalized | 0.0251 |
| original_glrlm_LongRunLowGrayLevelEmphasis | 0.0399 |
| original_glszm_GrayLevelNonUniformity | 0.0393 |
| original_glszm_GrayLevelNonUniformityNormalized | 0.0377 |
| original_glszm_HighGrayLevelZoneEmphasis | 0.0418 |
| original_glszm_LargeAreaEmphasis | 0.0070 |
| original_glszm_LargeAreaHighGrayLevelEmphasis | 0.0070 |
| original_glszm_LargeAreaLowGrayLevelEmphasis | 0.0362 |
| original_glszm_SizeZoneNonUniformity | 0.0024 |
| original_glszm_SizeZoneNonUniformityNormalized | 0.0213 |
| original_ngtdm_Coarseness | 0.0319 |
| original_ngtdm_Strength | 0.0198 |

**Supplemental Material 2**: 18 significant features with the p-value.


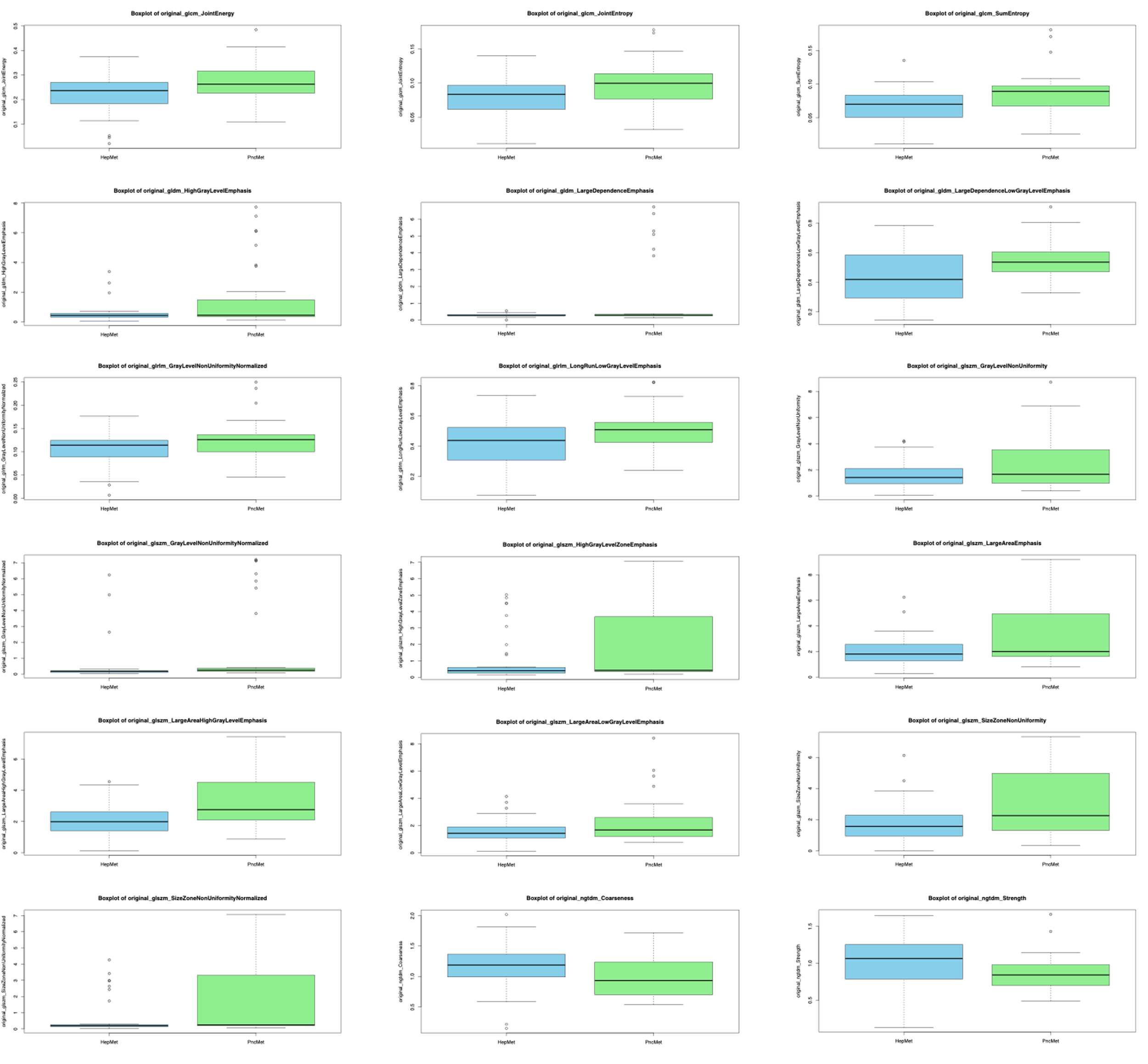


**Supplemental Material 3**: Boxplots of significant features.

**Supplemental Material 4:** Violin plot showing the distribution of lesion counts in colorectal and pancreatic cancer cohorts.
